# Chlorophyll Fluorescence and Biochemical Biomarkers Reveal Plasticizer Di-n-Butyl Phthalate-Induced Stress in *Azolla pinnata*

**DOI:** 10.3390/plants14233629

**Published:** 2025-11-28

**Authors:** Hari Dev Chaudhary, Upma Bhatt, Vineet Soni

**Affiliations:** Plant Bioenergetics and Biotechnology Laboratory, Department of Botany, Mohanlal Sukhadia University, Udaipur 313001, India; haridevscience1998@gmail.com (H.D.C.); bhattupma154@gmail.com (U.B.)

**Keywords:** antioxidant enzymes, biophysical parameters, oxidative stress, photosystem II efficiency, energy flux dynamics, lipid peroxidation, aquatic ecotoxicology

## Abstract

Phthalates, particularly di-n-butyl phthalate (DBP), are persistent plasticizers that pose serious ecological risks to aquatic environments. This study evaluated the phytotoxic effects of DBP on the aquatic fern *Azolla pinnata* through morphological, biochemical, and photosynthetic responses. Plants were exposed to graded DBP concentrations (0–10 mg/L) for 4 and 8 days. Increasing DBP levels caused visible symptoms including frond chlorosis, necrosis, and root inhibition. Biochemically, total chlorophyll content declined by up to 95%, while malondialdehyde (MDA) levels increased by approximately 300% at 10 mg/L, confirming severe lipid peroxidation. Antioxidant enzymes showed biphasic trends: superoxide dismutase (SOD) and catalase (CAT) activities rose under moderate stress but declined by ~73% and ~78%, respectively, at the highest concentration, indicating oxidative enzyme suppression. Chlorophyll fluorescence analysis revealed strong, dose-dependent inhibition of photosystem II efficiency, characterized by reduced performance indices (PIabs, PIcs) and quantum yields (фPo, фEo), alongside increased фDo and Fo/Fm, indicating enhanced energy dissipation and photoinhibition. Overall, DBP exposure disrupted oxidative balance and PSII function in *A. pinnata*, demonstrating its high sensitivity to phthalate toxicity and validating chlorophyll fluorescence as a rapid, non-invasive biomonitoring tool for assessing aquatic pollutant stress.

## 1. Introduction

Phthalates (PAEs), listed as priority pollutants by the U.S. Environmental Protection Agency (USEPA) and regulated by the European Commission, have raised significant environmental concerns due to their widespread occurrence and persistence [1]. These compounds, chemically defined as dialkyl or alkyl aryl esters of 1,2-benzenedicarboxylic acid, are categorized into high and low-molecular-weight phthalates (HMWP and LMWP) and are extensively used as plasticizers. In 2020, phthalates accounted for over 55% of global plasticizer consumption. Due to their ability to improve flexibility, transparency, and durability, PAEs have been incorporated into various polymeric and non-polymeric materials, especially plastics like polyethylene (PE), polypropylene (PP), polystyrene (PS), and polyvinyl chloride (PVC) [2,3].

Apart from industrial applications, phthalates are also present in consumer goods such as food packaging, flooring, cosmetics, and personal care products, including nail polish, moisturizers, soaps, and perfumes [4,5]. Among the most commonly used PAEs are Di(2-ethylhexyl) phthalate (DEHP), mainly associated with PVC products and food packaging; di-n-butyl phthalate (DBP), found in adhesives and cosmetics; benzyl butyl phthalate (BBP), used in flooring and paints; diethyl phthalate (DEP), prevalent in fragrances; and dimethyl phthalate (DMP), used in plastics and personal care items [6,7].

Environmental monitoring has detected PAE residues in air, rainwater, soil, sediments, and wastewater [8,9], a reflection of the increasing global use of plastics. Notably, around 40–50% of all industrial plasticizers are phthalates, with production growing by approximately six million metric tons annually [10,11]. These compounds, being non-covalently bonded to polymers, readily leach into environmental media such as water, soil, sediments, air, and food [10,12], raising concerns over long-term contamination and human exposure. Phthalate exposure has been linked to various health issues, particularly during pregnancy, such as fetal developmental abnormalities, cardiovascular diseases, diabetes, and hypertension [13,14]. Other health impacts include preterm births, miscarriage, insulin resistance, and endocrine disruption [15,16]. Human exposure occurs via ingestion, inhalation, and dermal contact, with phthalate metabolites commonly detected in urine, as well as in serum, saliva, and breast milk [17].

Environmental persistence of phthalates, particularly in soil due to plastic waste disposal, contributes to chronic ecological risks. These compounds negatively impact soil microbial activity and diversity [18,19], thereby impairing soil fertility and ecosystem functioning. Plants can absorb PAEs through roots and transport them to stems, leaves, and fruits, leading to reduced growth and productivity [20,21]. Furthermore, PAEs exhibit toxicity towards soil organisms such as earthworms and microbes by altering gene expression, which disrupts physiological processes and soil biodiversity [22,23]. Their persistence and bioaccumulative nature make them a concern for long-term environmental health and food chain contamination [24]. Dibutyl phthalate (DBP), a widely used phthalate in the plastics industry, has been designated a priority pollutant by the USEPA. DBP has been detected in various environmental compartments, including drinking water, air, dust, sediments, and soil [25,26]. It has been shown to interfere with biochemical pathways in plants, such as carotenoid biosynthesis, and has been linked to abnormal seedling development [27]. Toxicological studies have reported that DBP exposure impairs semen quality in males, increases breast cancer risk, and may lead to inflammation and pre-cancerous lesions following prenatal and lactational exposure [28,29,30]. Due to its mutagenic, teratogenic, and carcinogenic potential, DBP is categorized as a high-priority environmental toxic compound [31,32]. Given the increasing use of plastic and the resulting environmental burden of phthalates, there is a growing need to investigate their impact on plant systems. Since plants are indicators of environmental stress, physiological parameters such as germination rate, growth metrics, pigment concentration, and antioxidant activity serve as vital markers of pollutant toxicity [31,33,34]. Several studies on aquatic macrophytes and algae have demonstrated that exposure to DBP or related phthalates causes significant declines in pigment contents, growth, and induces oxidative stress, which are likely to be reflected in parameters such as chlorophyll fluorescence in your work. For instance, *Spirodela polyrhiza* plants exposed to increasing concentrations of DBP showed sharp reductions in chlorophyll content and soluble protein levels, coupled with elevated lipid peroxidation (measured via malondialdehyde, MDA) and increased activity of antioxidant enzymes (e.g., catalase, peroxidase) [35]. Similarly, the green alga *Chlorella vulgaris* exhibited a 24 h EC_50_ of ~4.95 mg/L for DBP, associated with a significant decrease in chlorophyll *a* and *b* content, illustrating impairment in photosynthetic pigment machinery [36]. These findings indicate that in aquatic plants, DBP exposure even at moderate levels can degrade pigments, reduce protein content, generate reactive oxygen species (ROS), damage cellular membranes, and by extension, likely reduce photosystem II performance (e.g., via decreases in Fv/Fm or other OJIP fluorescence parameters) well before visible morphological damage becomes severe. Therefore, this study focuses on evaluating the toxicological effects of di-n-butyl phthalate (DBP) on *A. pinnata*, with particular emphasis on biochemical, biophysical responses, photosynthetic efficiency, and physiological alterations induced by phthalate exposure. *A. pinnata* was selected as the test plant owing to its exceptional sensitivity to environmental contaminants and its well-established potential as a bioindicator of aquatic pollution [37]. As a free-floating water fern with a symbiotic association with *Anabaena azollae*, it plays a vital role in nitrogen fixation, nutrient cycling, and primary productivity in freshwater ecosystems [38]. These ecological functions make it highly responsive to stressors that disrupt photosynthesis and redox homeostasis. Moreover, *A. pinnata* exhibits rapid and measurable physiological and biochemical alterations such as changes in pigment content, antioxidant enzyme activity, and chlorophyll fluorescence under pollutant exposure, allowing it to serve as an early warning system for aquatic toxicity [39]. In this study, chlorophyll *a* fluorescence OJIP transients were employed as a non-invasive and highly sensitive diagnostic tool to detect early perturbations in photosystem II (PSII) function. This technique provides a rapid, cost-effective, and reproducible means of evaluating pollutant-induced stress in aquatic plants, offering significant practical relevance for environmental monitoring and ecotoxicological risk assessment. Therefore, this study aimed to elucidate the dose- and time-dependent effects of di-n-butyl phthalate (DBP) on photosystem II integrity and antioxidant defense mechanisms in *A. pinnata* using chlorophyll fluorescence transients (OJIP) and key biochemical biomarkers. This integrative approach provides novel insights into the mechanisms of phthalate-induced phytotoxicity and reinforces the value of *A. pinnata* as a bioindicator species for aquatic ecotoxicological assessment.

## 2. Materials and Methods

### 2.1. Plant Material and Growth Conditions

*Azolla pinnata* plants were obtained from the Ayad River located at Udaipur, India (coordinates 24°34′54″ N and 73°42′40″ E). Species confirmation was based on its characteristic morphology, including small, finely divided fronds that display shades ranging from green to reddish-brown. The plant exhibits a compact, branched stem with delicate roots extending into the water column. Leaves are arranged alternately, with a thick, chlorophyll-rich dorsal lobe above and a thinner, translucent ventral lobe beneath [40]. To ensure representativeness and minimize sampling bias, plants were collected using a random sampling approach from multiple locations within the river. To support vigorous vegetative growth, sample (River) water was supplemented weekly with a mineral mixture containing magnesium chloride, rock phosphate, and potassium in a 2:3:1 (*w*/*w*/*w*) proportion, at a concentration of 1 mg/L. After one week, healthy and uniform plants were selected and transferred to a controlled growth chamber for experimental use. Cultures were maintained in beakers containing 0.1× Hoagland medium [41] under controlled conditions of 25–28 °C, 70–75% relative humidity, and a 16:8 h light–dark photoperiod. Prior to DBP treatment, plants were acclimated under the same conditions for seven days to stabilize growth.

### 2.2. DBP Treatment Preparation and Application

Analytical-grade di-n-butyl phthalate (DBP, purity ≥ 98%) was obtained from Sigma-Aldrich (Banglore, India) and used without further purification. A concentrated stock solution (100 g L^−1^) was prepared in analytical-grade acetone and stored at 4 °C in amber glass bottles. For each experimental exposure, freshly prepared dilutions were made from the acetone stock immediately before application to minimize potential volatilization or adsorption losses. Test solutions were prepared by adding the appropriate volume of DBP stock to the nutrient medium supporting *A. pinnata* growth, yielding final concentrations of 0, 2, 4, 6, 8, and 10 mg/L. The selected concentration range was based on previous ecotoxicological studies involving DBP and aquatic plant systems [42], ensuring both environmental relevance and the ability to capture graded phytotoxic responses. All treatment solutions were freshly prepared from analytical-grade stock before each exposure to maintain nominal concentration accuracy. To maintain consistent solvent exposure, the final acetone concentration was standardized to 0.01% (*v*/*v*) across all treatments, including the solvent control. This level was chosen because concentrations ≤0.01% acetone have been widely reported to exert no measurable effects on plant growth, pigment content, or photosynthetic performance [43]. Following one week of acclimation under controlled laboratory conditions, *A. pinnata* fronds were assigned to experimental groups: control (no solvent), solvent control (0.01% acetone), and DBP treatments. Each condition was established in triplicate (*n* = 3). At the end of 4 and 8 days of exposure, fronds were harvested for biochemical and chlorophyll fluorescence analyses to assess the sub-lethal impacts of DBP.

### 2.3. Morphological Measurement

To evaluate the morphological variability of *A. pinnata* in response to DBP exposure, qualitative parameters including frond size, frond color, and overall morphological appearance were visually assessed. At the end of the 8th day exposure period, a single image of each specimen was captured using a Nikon D7500 DSLR camera (20.9 MP resolution; Nikon Corporation, Tokyo, Japan) positioned at a fixed distance of 70 cm under dispersed natural light conditions to minimize glare and shadow.

### 2.4. Determination of Total Chlorophyll Content

Chlorophyll content was analysed by optimising the method given by Su et al. [44]. Fronds weighing 300 mg treatments were collected from the control and on the 0th and 4th day. The samples were macerated and homogenised in 10 mL of 80% chilled acetone, followed by incubation in the dark for 12 h at 24 °C. To determine the amount of chlorophyll, the plant extract was centrifuged for 10 min at 4 °C and 4000× *g*. Using the supernatant, Chlorophyll content was determined at 645 and 663 nm using spectrophotometry (Analytikjena^®^Specord 200, Jena, Germany) with a 1 cm path length quartz cuvette. The concentration of total chlorophyll $\left({mg\cdot g}^{-1}\right)$ was calculated by the following equations [45].

$$Total\;Chlorophyll\left({\mu g\cdot g}^{-1}\right)\;=\;20.31\;\times \;{OD}_{645}\;+\;8.05\;\times \;{OD}_{663}$$

$$Total\;Chlorophyll\left({mg\cdot g}^{-1}\right)\;=\;\frac{Chl\;\left({\mu g\cdot g}^{-1}\right)\;\times \;V}{W\;\times \;1000}$$

where *W* is the sample’s fresh tissue weight (g) and *V* is the extraction solvent volume (mL) in each sample. All measurements were performed in triplicate (*n* = 3).

### 2.5. Lipid Peroxidation (MDA) and Hydrogen Peroxide Quantification

The extent of lipid peroxidation was determined by quantifying malondialdehyde (MDA) following the thiobarbituric acid (TBA) method [46]. Frond tissue (100 mg fresh weight) was homogenized in 3 mL of 0.1% (*w*/*v*) trichloroacetic acid (TCA) at 4 °C. The homogenate was centrifuged at 4500× *g* for 15 min at 4 °C, and 1 mL of the clear supernatant was mixed with an equal volume of 20% TCA containing 0.5% TBA. The reaction mixture was boiled for 25 min in a water bath and then rapidly cooled on ice. After centrifugation to remove any precipitate, the absorbance of the supernatant was recorded at 532 nm and 600 nm using a UV–Vis spectrophotometer. Blank corrections were applied by subtracting the absorbance of reagent blanks and the 600 nm turbidity correction from the 532 nm readings before calculations. The concentration of thiobarbituric acid-reactive substances (TBARS), primarily MDA and its related endoperoxides, was determined using an extinction coefficient of 155 mM^−1^ cm^−1^ and expressed on a fresh-weight basis as nmol MDA g^−1^ FW. Hydrogen peroxide (H_2_O_2_) levels were quantified following Velikova et al. [47]. TCA extracts were mixed with phosphate buffer (pH 7.0) and potassium iodide (KI), incubated in darkness for 2 h with intermittent vortexing, and the absorbance was measured at 390 nm. H_2_O_2_ concentrations were calculated from a standard curve and expressed as µmol g^−1^ FW. All measurements were performed in triplicate (*n* = 3).

### 2.6. Estimation of Antioxidant Enzyme Activities (SOD, CAT)

For antioxidant enzyme extraction, 100 mg of *A. pinnata* tissue was homogenized in 1.2 mL of 50 mM potassium phosphate buffer (pH 7.8) containing 0.1 mM EDTA at 4 °C. After sequential centrifugations (15,000× *g*, 20 min), pooled supernatants were used to determine SOD and CAT activities, with three replicates per time point [48].

**Catalase (CAT; EC 1.11.1.6)** Activity was assayed according to the principle of Aebi [49], wherein the enzymatic breakdown of hydrogen peroxide is tracked by monitoring absorbance changes at 240 nm. For the assay, the reaction system (3.0 mL total volume) comprised 2.8 mL of 50 mM potassium phosphate buffer (pH 7.8), 80 µL of freshly prepared 500 mM H_2_O_2_ solution, and 120 µL of enzyme extract. The decrease in absorbance at 240 nm, reflecting substrate depletion, was recorded over the reaction period and employed to calculate CAT activity (mmol min^−1^ mg protein^−1^).

**Superoxide Dismutase (SOD; EC 1.15.1.1)** Activity was assayed following Beyer & Fridovich (1987) [50] by evaluating inhibition of nitroblue tetrazolium (NBT) photoreduction at 560 nm in the presence of riboflavin, using a mixture of 50 mM phosphate buffer (pH 7.8), 0.1% Triton X-100, 0.1 mM EDTA, 0.06 mM NBT, 10 mM methionine, 2 µM riboflavin, and enzyme extract; one unit corresponded to the enzyme quantity producing 50% inhibition of NBT reduction, expressed as U g^−1^ FW.

### 2.7. Fast Chlorophyll a Fluorescence Kinetics (ChlF)

The photochemical activity of PSII was assessed through the JIP-test, a refined chlorophyll fluorescence (ChlF) technique that deciphers the rapid O–J–I–P induction curve to extract biophysical parameters of energy conversion [51,52,53]. This approach dissects the successive phases of fluorescence kinetics, enabling evaluation of light absorption, excitation capture at the reaction centre (RC), electron transport dynamics, and dissipation of excess energy within the photosynthetic machinery. Fluorescence was measured using a Plant Efficiency Analyser (Hansatech Instruments, King’s Lynn, UK) after 1 h of dark adaptation to achieve full oxidation of PSII RCs. A saturating pulse of red light (λ = 650 nm, 3000 µmol photons m^−2^ s^−1^) was applied, and fluorescence emission from a 4 mm frond area was recorded over 1 s. The data acquisition was performed at an integration time of 10 µs with a gain setting of 1×, and the repetition rate was set to one measurement per second. Each measurement represented the average of three technical replicates per sample (*n* = 3 biological replicates). The OJIP transients were normalized between F_0_ and Fm, expressing variable fluorescence as (Ft − F_0_)/(Fm − F_0_) prior to JIP-test analysis to ensure comparability across treatments. The initial O-step (~50 µs) indicates minimal fluorescence (F_0_) with all RCs open, followed by the J-step (~2 ms) and I-step (~60 ms), which reflect sequential reduction in electron acceptors. The transient reaches its peak (P-step) at maximal fluorescence (Fm), corresponding to complete RC closure. The JIP-test translates these transients into quantitative descriptors that partition absorbed energy between photochemistry and non-photochemical losses. Derived parameters include fluxes per RC and per excited cross-section, quantum efficiencies of primary and downstream electron transport, and performance indices summarizing overall PSII competence [51,52,54,55,56,57]. All treatments were analysed in triplicate on both the 4th and 8th day to ensure consistency and statistical robustness. A compiled dataset of the calculated ChlF parameters, including energy fluxes, efficiency ratios, and performance indices, is presented in Table 1 [51,52,57].

### 2.8. Statistical Analysis

Statistical analyses were performed using Origin Pro 2025. One-way ANOVA was used to assess the significance of treatment effects, followed by Tukey’s HSD post hoc test for pairwise comparisons when significant differences were found. A significance level of *p* ≤ 0.05 was applied, and only statistically significant results are illustrated in the figures. Data are expressed as mean ± standard deviation (SD), based on a minimum of three independent biological replicates. In addition to univariate tests, multivariate methods were used to analyze relationships among chlorophyll fluorescence parameters. Principal Component Analysis (PCA) in Origin Pro 2025 was employed to visualize grouping patterns and treatment effects, helping to identify key stress-responsive traits. A Pearson correlation heatmap was also created using Origin Pro 2025 to reveal positive and negative associations among parameters, enhancing the interpretation of trends under DBP exposure.

## 3. Result

### 3.1. Morphological Changes

**Figure 1:**
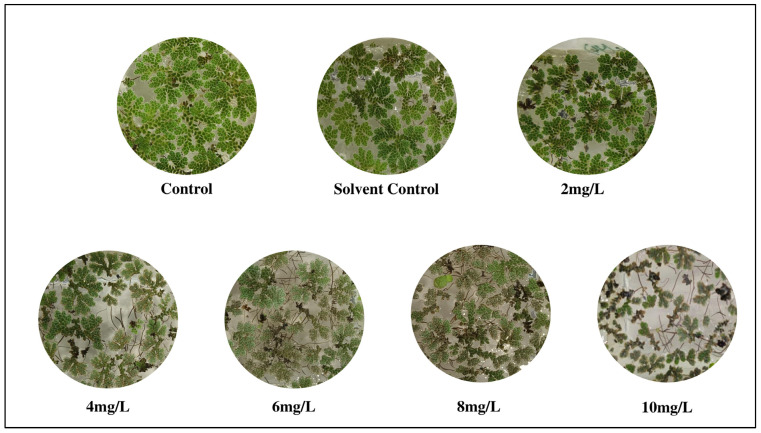
Morphological changes in *Azolla pinnata* after 8 days of DBP exposure, showing dose-dependent chlorosis and frond degradation.

The visible morphological alterations (Figure 1) in *A. pinnata* fronds following 8 days of exposure to different DBP concentrations (0, 2, 4, 6, 8, and 10 mg/L). Plants in the control and solvent control (0.01% acetone) treatments exhibited healthy, bright green fronds with compact and vigorous growth, indicating normal physiological conditions. At 2 mg/L, fronds showed slight chlorosis at the margins, but overall morphology remained largely unaffected. With increasing DBP concentrations (4–6 mg/L), fronds progressively exhibited chlorosis, partial browning, and fragmentation, suggesting early signs of oxidative and structural stress. At higher concentrations (8–10 mg/L), severe morphological deterioration was evident, characterized by bleaching and disintegration of frond structure, reflecting advanced phytotoxic damage. These visible symptoms confirm the concentration-dependent toxic effects of DBP on *A. pinnata* morphology.

### 3.2. Effect of DBP on Biochemical Parameters of A. pinnata

#### 3.2.1. Total Chlorophyll Content

**Figure 2:**
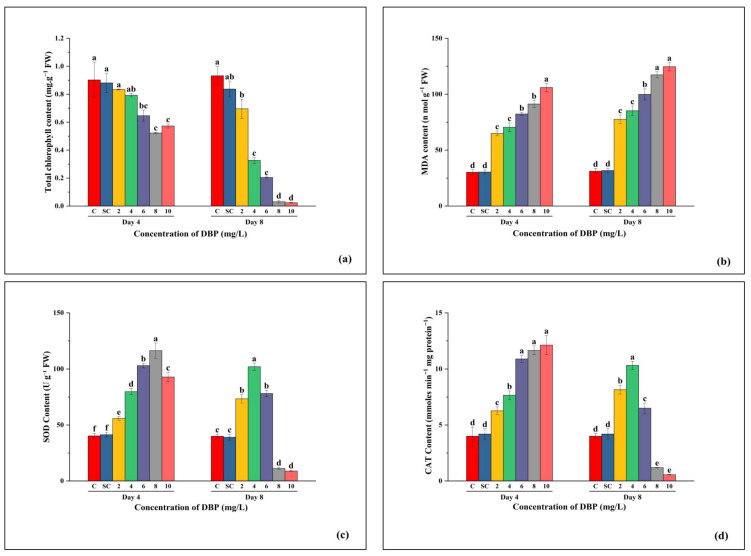
Effects of di-n-butyl phthalate (DBP) on (**a**) total chlorophyll, (**b**) malondialdehyde (MDA), (**c**) superoxide dismutase (SOD), and (**d**) catalase (CAT) contents in *A. pinnata* after 4 and 8 days of exposure at 0–10 mg/L DBP. C = control, SC = solvent control. Different letters above bars indicate significant differences among treatments at *p* < 0.05.

On Day 4, total chlorophyll content declined progressively with increasing DBP concentrations compared to the control. The control (C) and solvent control (SC) maintained the highest chlorophyll levels, with no significant difference between them. Reductions became evident from 4 mg/L onward, with the lowest values at 8 mg/L. By Day 8, chlorophyll content further decreased in all DBP treatments, showing a steep decline at 8 and 10 mg/L, where values dropped close to zero, whereas C and SC remained significantly higher and unaffected (Figure 2a).

#### 3.2.2. MDA Content

MDA content increased consistently with rising DBP concentrations. On Day 4, C and SC exhibited the lowest values with no significant difference between them, while the treated groups showed a dose-dependent rise, peaking at 10 mg/L. By Day 8, MDA levels were higher overall, with the most pronounced increase at 8 and 10 mg/L. C and SC remained low and stable (Figure 2b).

#### 3.2.3. SOD Activity

On Day 4, SOD activity increased with DBP concentration up to 8 mg/L, after which it slightly declined at 10 mg/L. C and SC values were similar and lower than the 2–6 mg/L treatments. On Day 8, SOD activity was reduced across all DBP treatments compared to Day 4, with the highest activity observed at 4 mg/L. The lowest SOD activity occurred at 10 mg/L, while C and SC remained unchanged and were not different from each other (Figure 2c).

#### 3.2.4. CAT Activity

On Day 4, the CAT activity rose steadily with DBP concentration, reaching its maximum at 8 and 10 mg/L. C and SC showed no difference and maintained the lowest values. On Day 8, CAT activity peaked at 4 mg/L, followed by a decline at 6 mg/L and a sharp drop at 10 mg/L. As in other parameters, no difference was observed between C and SC (Figure 2d).

### 3.3. Photosynthetic Performance

#### 3.3.1. OJIP Chlorophyll Fluorescence Transients of *A. pinnata* Under DBP Exposure

**Figure 3:**
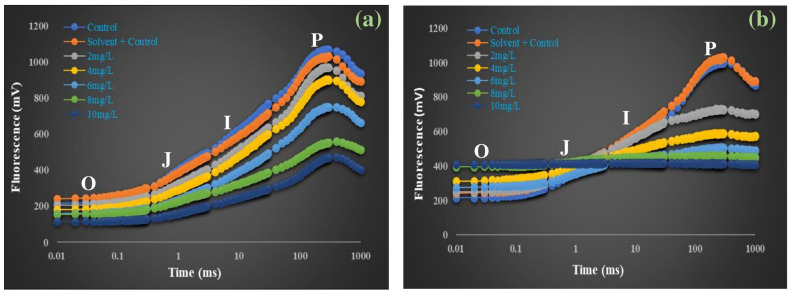
Chlorophyll *a* fluorescence transient (OJIP curve) of *A. pinnata* exposed to di-n-butyl phthalate (DBP) at 0–10 mg/L for (**a**) 4 days and (**b**) 8 days.

The OJIP fluorescence transient reflects the sequential reduction in electron carriers in PSII, where the O step corresponds to minimal fluorescence (Fo), J and I indicate progressive reduction in Q_A_ and plastoquinone pool, and the P step represents the maximum fluorescence (Fm). Alterations in these phases, therefore, provide insight into PSII integrity and electron transport efficiency. On Day 4 (Figure 3a), all treatments displayed the typical O–J–I–P fluorescence rise. The control (C) and solvent control (SC) curves overlapped completely, showing the highest fluorescence intensities at all steps with no difference between them. In DBP-treated plants, fluorescence intensity decreased progressively with increasing concentration. At 2 mg/L, the reduction was slight, while at 4 and 6 mg/L, a noticeable lowering of the J, I, and P steps was observed. The decline became more pronounced at 8 mg/L and was most severe at 10 mg/L, where the rise beyond the J step was strongly suppressed, and the P peak was lowest compared to all other treatments. On Day 8 (Figure 3b), the separation between treatments became more distinct. Control and SC again showed overlapping curves with consistently high fluorescence intensities and no difference between them. In DBP treatments, the reduction was stronger than on Day 4. At 2 and 4 mg/L, decreases at the J, I, and P steps were clear but moderate, while 6 mg/L showed a sharper decline. At 8 and 10 mg/L, the curves displayed a distinct pattern: the O step (Fo) showed a clear increase compared to Day 4, while the P step (Fm) was drastically reduced. As a result, beyond the J point, the rise toward P was almost absent, producing a nearly straight line between J, I, and P. Compared with Day 4, this flattening was much more pronounced, indicating a severe suppression of maximal fluorescence under prolonged exposure.

#### 3.3.2. Effects of DBP on Biophysical Parameters

**Figure 4:**
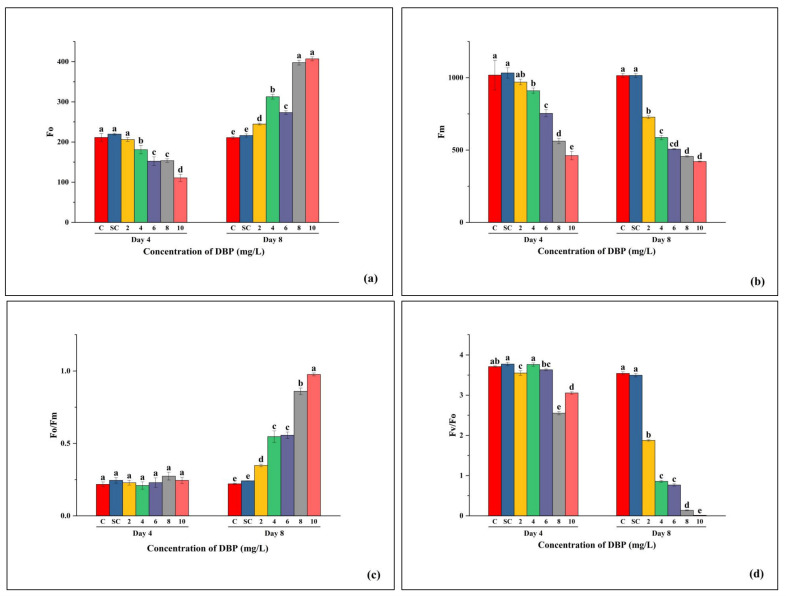
Effects of di-n-butyl phthalate (DBP) on chlorophyll fluorescence parameters of *A. pinnata*: (**a**) Fo, (**b**) Fm, (**c**) Fo/Fm, and (**d**) Fv/Fo after 4 and 8 days of exposure at 0–10 mg/L DBP. C = control, SC = solvent control. Different letters above bars indicate significant differences among treatments at *p* < 0.05.

Fo, which represents the basal fluorescence when PSII reaction centers are fully open (Figure 4a), showed no difference between C and SC. At Day 4, Fo decreased with increasing DBP concentration, indicating partial inhibition of PSII centers. By Day 8, Fo increased significantly at higher concentrations (8–10 mg/L), reflecting PSII damage and reaction center closure due to stress. Fm, reflecting the maximum fluorescence when all PSII reaction centers are closed (Figure 4b), remained stable in C and SC. At Day 4, Fm declined progressively with DBP dose, and this reduction was even stronger by Day 8, particularly at 6–10 mg/L. The decrease in Fm indicates impaired energy transfer capacity and reduced maximum photochemical efficiency under DBP stress. The Fo/Fm ratio, which provides an estimate of PSII energy loss (Figure 4c), was unaffected in C and SC. At Day 4, values were close to controls across concentrations, with only slight increases at 8–10 mg/L. By Day 8, Fo/Fm rose sharply in a concentration-dependent manner, with maximum values at 10 mg/L, suggesting enhanced non-photochemical quenching and energy dissipation. Fv/Fo, a parameter describing the maximum efficiency of the water-splitting complex on the donor side of PSII (Figure 4d), showed no difference between C and SC. At Day 4, values remained stable up to 4 mg/L but decreased at 6–10 mg/L. By Day 8, a sharp concentration-dependent decline was observed, with near-complete loss of activity at 8–10 mg/L, confirming severe inhibition of PSII photochemistry.

#### 3.3.3. Leaf (Phenomenological) Model

**Figure 5:**
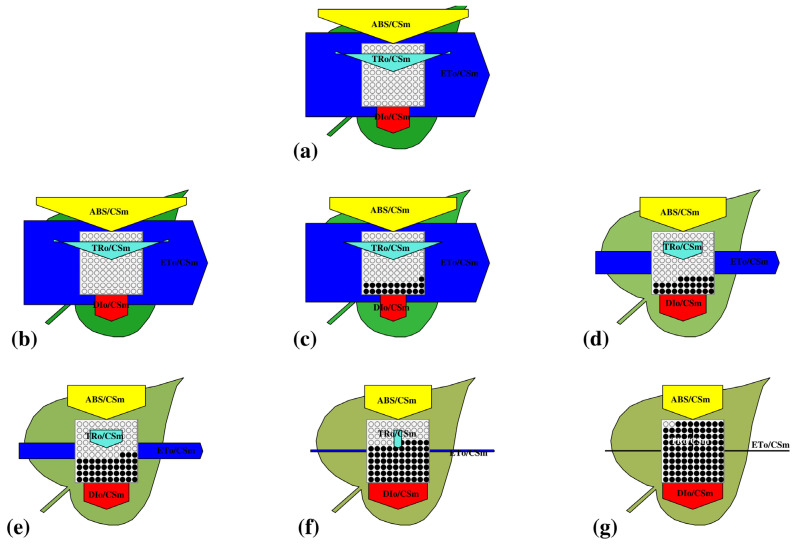
Energy pipeline leaf models illustrating the effects of di-n-butyl phthalate (DBP) on *A. pinnata*. Parameters represent absorption (ABS/CSm), trapping (TRo/CSm), electron transport (ETo/CSm), and dissipation (DIo/CSm) after exposure to different DBP concentrations. Where (**a**) is control, (**b**) solvent-control, and (**c**–**g**) represents exposure of 2, 4, 6, 8 and 10 mg/L DBP concentrations respectively.

The changes in energy fluxes (ABS/CSm, TRo/CSm, ETo/CSm, DIo/CSm) under increasing DBP concentrations (Figure 5a–g). In both C and SC, flux distributions remained balanced, with no apparent difference between them. At 2 mg/L DBP, only a minor shift in fluxes was observed, with energy absorption and trapping similar to those of the controls. With further increase in concentration (4–6 mg/L), energy trapping (TRo/CSm) and electron transport (ETo/CSm) gradually declined, accompanied by a noticeable increase in energy dissipation (DIo/CSm). At the highest concentrations (8–10 mg/L), flux maps showed severe alterations, with a marked reduction in TRo/CSm and ETo/CSm, while DIo/CSm strongly increased, indicating enhanced energy loss.

#### 3.3.4. Energy Flux Parameters per Cross Section

**Figure 6:**
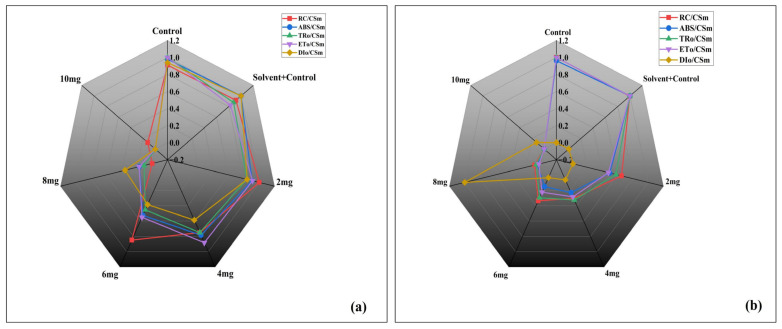
Radar plots showing the effects of di-n-butyl phthalate (DBP) on photosynthetic energy flux parameters of *A. pinnata* after (**a**) 4 days and (**b**) 8 days of exposure at 0–10 mg/L. Parameters include reaction centers per cross-section (RC/CSm), absorption flux (ABS/CSm), trapping flux (TRo/CSm), electron transport flux (ETo/CSm), and dissipation flux (DIo/CSm).

At Day 4 (Figure 6a), both C and SC exhibited nearly identical flux values across all parameters, confirming no difference between them. Low DBP concentrations (2 mg/L) caused negligible deviation from controls, while moderate concentrations (4–6 mg/L) led to a decline in RC/CSm, ABS/CSm, TRo/CSm, and ETo/CSm, together with a relative rise in DIo/CSm. At higher concentrations (8 and 10 mg/L), all functional flux parameters were strongly suppressed, whereas DIo/CSm showed a marked increase, highlighting enhanced energy dissipation. At Day 8 (Figure 6b), deviations were more pronounced than on Day 4. Controls (C and SC) again remained unaffected and overlapped closely. Even at 2 mg/L, energy flux parameters began to decline, and DIo/CSm showed a clear increase compared to Day 4. Higher concentrations (6–10 mg/L) showed drastic reductions in TRo/CSm, ETo/CSm, ABS/CSm, and RC/CSm, while DIo/CSm expanded prominently, confirming progressive impairment of PSII energy distribution with exposure time and dose.

#### 3.3.5. Changes in Kn and Kp of *A. pinnata* Under DBP Exposure

**Figure 7:**
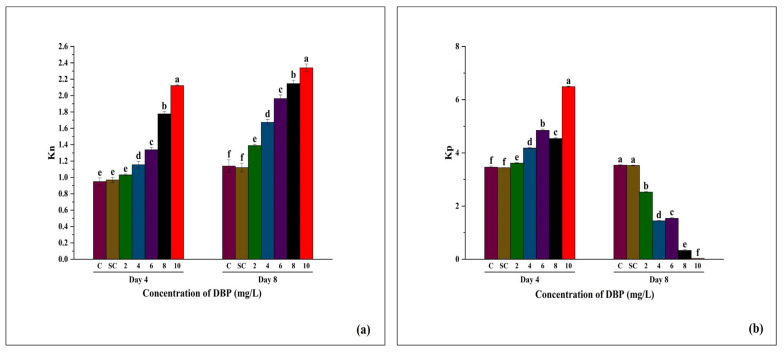
Effects of di-n-butyl phthalate (DBP) on photosynthetic performance indices of *A. pinnata*: (**a**) Kn (non-photochemical de-excitation constant) and (**b**) Kp (photochemical de-excitation constant) after 4 and 8 days of exposure at 0–10 mg/L DBP. C = control, SC = solvent control. Different letters above bars indicate significant differences among treatments at *p* < 0.05.

Kn (non-photochemical de-excitation constant) reflects the extent of energy dissipation as heat (Figure 7a), while Kp (photochemical rate constant) indicates the efficiency of energy utilization in PSII photochemistry (Figure 7b). No significant difference was observed between C and SC on both days. At Day 4, Kn increased progressively with increasing DBP concentrations, reaching the highest value at 10 mg/L. By Day 8, this trend became more pronounced, with significantly higher Kn values recorded in all DBP treatments compared to controls, and the strongest effect again at 10 mg/L. C and SC showed similar values at both time points. At Day 4, Kp gradually increased with rising DBP concentrations, peaking at 10 mg/L. However, at Day 8, a clear opposite pattern was observed: Kp declined progressively with increasing concentrations, with the lowest value recorded at 10 mg/L, while C and SC maintained consistently higher levels.

#### 3.3.6. Quantum Yield of *A. pinnata* Under DBP Exposure

**Figure 8:**
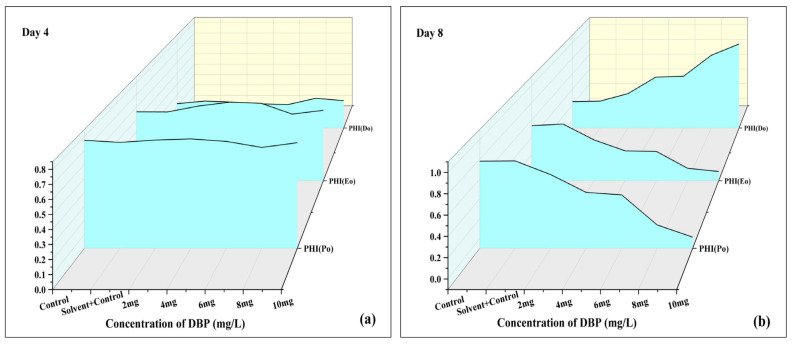
Effects of di-n-butyl phthalate (DBP) on quantum yield parameters of *A. pinnata* after (**a**) 4 days and (**b**) 8 days of exposure at 0–10 mg/L. Parameters: PHI(Po) (maximum quantum yield of PSII), PHI(Eo) (quantum yield for electron transport), PHI(Do) (quantum yield for energy dissipation).

PHI(Po) represents the maximum photochemical efficiency of PSII at the onset of illumination, PHI(Eo) reflects the efficiency of electron transport beyond Q_A_, and PHI(Do) indicates the fraction of absorbed energy dissipated as heat. Together, these parameters provide insights into the balance between energy utilization and dissipation under stress conditions. At Day 4 (Figure 8a), no difference was observed between C and SC. The maximum photochemical efficiency (PHI(Po)), which reflects the efficiency of primary photochemistry, and PHI(Eo), related to electron transport beyond Q_A_, remained relatively stable across 2–6 mg/L, with only slight decreases at higher concentrations. In contrast, PHI(Do), representing energy dissipation as heat, increased progressively with DBP concentration, with a clear rise at 8 and 10 mg/L. This suggests that under short-term exposure, PSII photochemistry was mostly maintained, but more absorbed energy was diverted to dissipation at higher stress levels. At Day 8 (Figure 8b), at this stage, stronger effects of DBP were evident. C and SC again showed stable values, indicating no difference between them. PHI(Po) and PHI(Eo) declined in a concentration-dependent manner, showing reduced photochemical efficiency and impaired electron transport, particularly at 8 and 10 mg/L. Conversely, PHI(Do) increased sharply with DBP concentration, with the highest dissipation at 10 mg/L. These results highlight a shift from efficient energy utilization to increased non-photochemical quenching under prolonged DBP exposure.

#### 3.3.7. Photosynthetic Performance Indices and Primary and Secondary Photochemistry of *A. pinnata* Under DBP Exposure

**PI(abs):**

**Figure 9:**
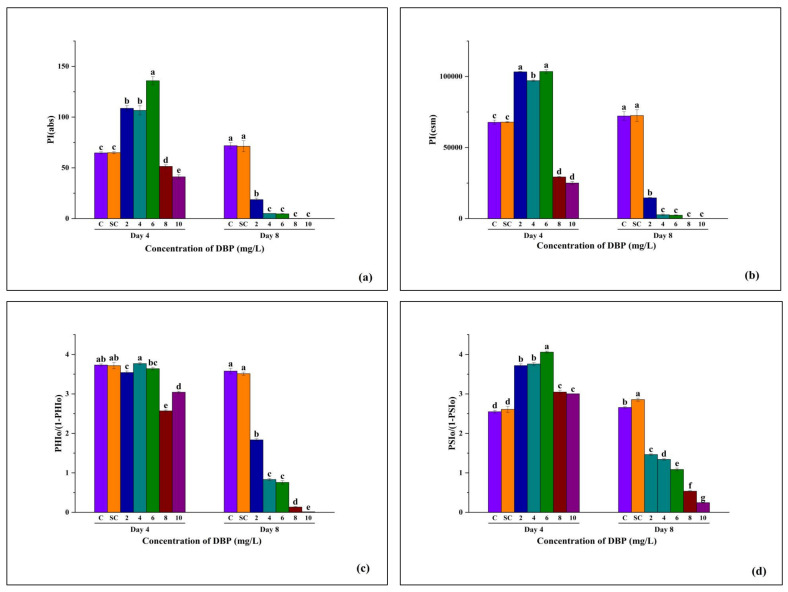
Effects of di-n-butyl phthalate (DBP) on performance indices of *A. pinnata* after 4 and 8 days of exposure at 0–10 mg/L: (**a**) PIabs (performance index based on absorption), (**b**) PIcs (performance index per cross-section), (**c**) PHI/(1 − PHIo) (quantum yield ratio for electron transport to PSI acceptors), and (**d**) PSIo/(1 − PSIo) (efficiency ratio for electron transfer beyond Q_A_^−^). C = control, SC = solvent control. Different letters above bars indicate significant differences among treatments at *p* < 0.05.

PI(abs), which reflects the overall performance index based on absorption, showed no difference between C and SC. At Day 4, PI(abs) increased slightly at 2–6 mg/L but then declined sharply at higher concentrations, with the lowest value at 10 mg/L. By Day 8, PI(abs) dropped drastically under all DBP treatments, with complete suppression at 4–10 mg/L, while C and SC remained stable (Figure 9a).

**PI(cs):**

PI(cs), representing performance index per cross-section, followed a similar pattern. On Day 4, moderate concentrations (2–6 mg/L) maintained higher PI(cs) than controls, but a steep decline was observed at 6–10 mg/L. At Day 8, PI(cs) values in DBP treatments were severely reduced in a concentration-dependent manner, reaching near-zero at 8 and 10 mg/L, whereas C and SC retained high values (Figure 9b).

**PHIo/(1–PHIo):**

This parameter, indicating the balance between photochemical activity and energy not used in photochemistry, showed no variation between C and SC. On Day 4, values remained stable at 2–6 mg/L but decreased significantly at 8 and 10 mg/L. By Day 8, a clear decline was evident across all treatments, with the steepest reduction at higher (8–10 mg/L) concentrations (Figure 9c).

**PSIo/(1–PSIo):**

This index reflects the balance between active PSI function and energy loss. At Day 4, PSIo/(1–PSIo) increased slightly at 2–6 mg/L, remained stable at 8 mg/L, and declined at 8–10 mg/L. At Day 8, values decreased progressively with increasing DBP concentration, reaching their minimum at 10 mg/L, while C and SC retained significantly higher values (Figure 9d).

### 3.4. Multivariate Analysis

#### 3.4.1. Principal Component Analysis (PCA) of Chlorophyll Fluorescence Parameters in *A. pinnata* Under DBP Stress

**Figure 10:**
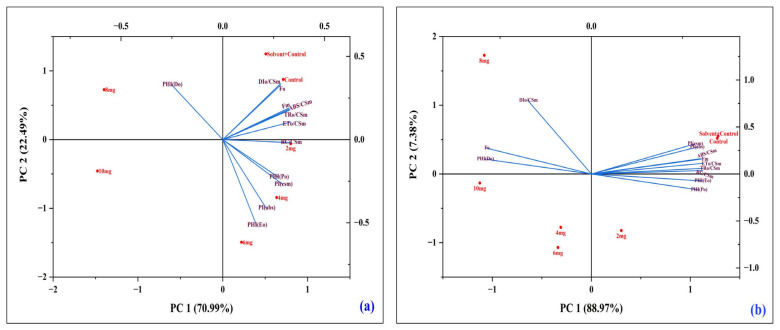
Principal component analysis (PCA) of chlorophyll fluorescence parameters in *A. pinnata* exposed to di-n-butyl phthalate (DBP) for (**a**) 4 days and (**b**) 8 days at 0–10 mg/L.

Principal Component Analysis revealed distinct clustering between treatments and controls. At Day 4 (Figure 10a), C and SC grouped with parameters reflecting efficient photochemistry (PI values, TRo/CSm, ETo/CSm), whereas 8–10 mg/L treatments separated strongly, associated with Fo and PHI(Do). By Day 8 (Figure 10b), separation became more pronounced: higher concentrations (6–10 mg/L) clustered with Fo and PHI(Do), while C and SC remained with absorption and performance indices. This confirmed progressive DBP-induced impairment of PSII over time, characterized by enhanced energy dissipation and reduced photochemical performance.

#### 3.4.2. Correlation Analysis of Chlorophyll Fluorescence and Performance Parameters Under DBP Exposure

**Figure 11:**
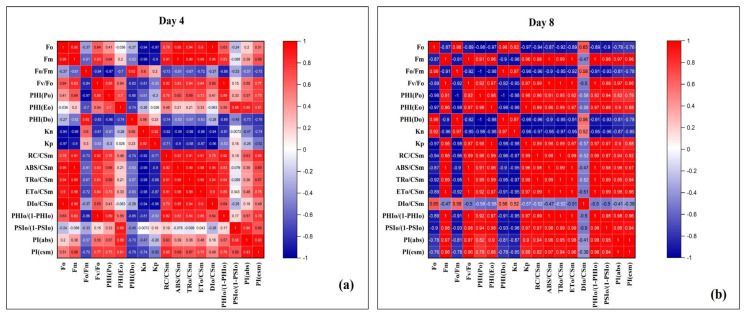
Correlation heatmaps of chlorophyll fluorescence parameters in *A. pinnata* exposed to di-n-butyl phthalate (DBP) for (**a**) 4 days and (**b**) 8 days. Parameters include Fo (minimum fluorescence), Fm (maximum fluorescence), quantum yields (PHI(Po), PHI(Eo), PHI(Do), performance indices (PIabs, PIcs), and energy fluxes (RC/CSm, ABS/CSm, TRo/CSm, ETo/CSm, DIo/CSm).

Correlation heatmaps showed distinct patterns between Day 4 and Day 8. At Day 4 (Figure 11a), most photochemical parameters such as Fm, Fv/Fo, ABS/CSm, TRo/CSm, and ETo/CSm were positively correlated with performance indices (PI(abs), PI(cs)), indicating coordinated PSII activity under short-term stress. Fo and PHI(Do) showed negative correlations with efficiency parameters, suggesting that increased minimal fluorescence and dissipation were linked with reduced photochemical stability, while Kp correlated positively with PI indices. By Day 8 (Figure 11b), the correlations became stronger and more polarized, with Fo, DIo/CSm, and PHI(Do) showing strong negative associations with PI(abs), PI(cs), and Fv/Fo, confirming that higher energy dissipation was directly linked with reduced PSII efficiency. In contrast, Fm, ABS/CSm, TRo/CSm, and ETo/CSm maintained strong positive correlations with performance indices, indicating that efficient energy trapping and transport supported better PSII performance even under prolonged DBP exposure.

## 4. Discussion

The present study demonstrates that exposure to DBP significantly alters the biochemical photosynthetic performance and chlorophyll fluorescence characteristics of *A. pinnata*, with the severity of effects depending on concentration and exposure duration. While control (C) and solvent control (SC) plants remained unaffected, DBP treatments progressively impaired PSII activity, particularly at higher concentrations and longer exposure.

### 4.1. DBP-Induced Oxidative Stress and Antioxidant Enzyme Response

The biochemical responses of *A. pinnata* under DBP exposure suggest that oxidative stress is a central mechanism of toxicity. A progressive reduction in total chlorophyll content with increasing concentrations and prolonged exposure indicates disruption of pigment biosynthesis and/or enhanced degradation. Such chlorophyll loss is a well-documented effect of phthalates and other xenobiotics in aquatic plants and algae, where oxidative damage to chloroplasts and inhibition of photosynthetic enzyme systems reduce light-harvesting capacity and carbon assimilation [35,36]. This pigment decline, therefore, reflects the vulnerability of the photosynthetic apparatus to DBP stress.

In parallel, malondialdehyde (MDA) content increased in a concentration- and time-dependent manner, indicating enhanced lipid peroxidation and membrane injury. MDA is a widely used biomarker of oxidative damage, and its elevation under DBP stress confirms that ROS accumulation exceeded the detoxification capacity of the antioxidant system [58,59,60]. This damage to membrane lipids likely underlies the impairment of chloroplast structure and function, further contributing to pigment loss and reduced photosynthetic performance [61]. The activities of antioxidant enzymes revealed a biphasic pattern characteristic of oxidative stress responses. Superoxide dismutase (SOD) activity increased under moderate stress, reflecting activation of the first line of defense against superoxide radicals, but declined at the highest dose and after prolonged exposure, suggesting enzyme inactivation or exhaustion [62,63]. Catalase (CAT) activity also rose initially, consistent with its role in detoxifying hydrogen peroxide generated by SOD, but later declined sharply at higher DBP concentrations, indicating that antioxidant defenses were overwhelmed. Similar transient stimulation followed by inhibition of antioxidant enzymes has been reported under phthalate and heavy metal stress in other aquatic macrophytes [64,65]. Collectively, these patterns indicate that DBP triggers oxidative stress by generating excessive ROS, which initially induces antioxidant defenses but ultimately suppresses them under severe or prolonged exposure. The imbalance between ROS production and antioxidant capacity leads to membrane lipid peroxidation, chlorophyll degradation, and progressive loss of photosynthetic efficiency. These findings align with previous reports demonstrating that phthalates impair cellular redox homeostasis, chloroplast integrity, and overall plant vitality [66,67,68]. From an ecological perspective, such biochemical impairment has important consequences. *A. pinnata* contributes to nitrogen fixation, nutrient cycling, and primary productivity in aquatic ecosystems. DBP-induced reductions in chlorophyll and antioxidant function could therefore diminish its growth, nitrogen-fixing ability, and ecological services, with cascading impacts on freshwater ecosystem stability [69,70]. The observed responses highlight the sensitivity of *A. pinnata* to phthalate stress and reinforce the utility of biochemical markers such as chlorophyll, MDA, and antioxidant enzymes for assessing pollutant toxicity in aquatic plants.

### 4.2. Distortion of OJIP Fluorescence Transients and PSII Photochemical Impairment

OJIP fluorescence transients are widely recognized as highly sensitive probes of PSII activity, reflecting the sequential reduction in electron carriers along the thylakoid electron transport chain [52,71]. In the present study, DBP exposure progressively distorted the OJIP fluorescence transients, particularly at higher concentrations and longer durations, indicating cumulative impairment of PSII function. The increase in minimal fluorescence (Fo) and concurrent decline in maximal fluorescence (Fm) suggest partial inactivation of PSII reaction centers, consistent with structural damage to the D1 protein and oxygen-evolving complex [72,73,74]. Suppression of the J–I–P phases further revealed a bottleneck in electron transport beyond Q_A_, including restricted plastoquinone (PQ) reduction and impaired PSI acceptor activity [51,55,75,76]. These alterations indicate a shift from efficient photochemistry to limited electron flow and enhanced non-photochemical dissipation. By Day 8, the effects intensified, with Fo further elevated and the I–P phase nearly absent at 8–10 mg/L, suggesting cumulative oxidative damage on both donor and acceptor sides of PSII. Such damage likely results from excessive reactive oxygen species (ROS) generation and lipid peroxidation of thylakoid membranes, which compromise PQ mobility and disrupt redox homeostasis [77,78,79]. The pattern of elevated Fo and suppressed Fm observed here aligns with previous reports of phthalate-induced photochemical inhibition in aquatic macrophytes and algae [80,81,82,83,84,85,86,87,88,89,90]. Similar trends were reported under di(2-ethylhexyl) phthalate (DEHP) exposure in *Lemna minor*, where PSII inactivation and ROS accumulation led to reduced chlorophyll fluorescence and electron transport [91], as well as in DBP-treated algal species showing comparable declines in photochemical efficiency [42]. These fluorescence impairments indicate a substantial reduction in the quantum yield of electron transport, directly translating to decreased photosynthetic carbon assimilation. For *A. pinnata*, a nitrogen-fixing fern critical to nutrient cycling and water quality regulation, such declines may lead to reduced growth and diminished ecological performance [80,81,82,83,84,85,86,87,88,89,90]. The fact that these effects were evident at the chlorophyll fluorescence level prior to visible symptoms further supports the OJIP test as an early, non-invasive biomarker of phthalate-induced PSII dysfunction [80,81,82,83,84,85,86,87,88,89,90].

### 4.3. Biophysical Disruption of PSII Function and Energy Flux Imbalance

The transient decrease in Fo observed on Day 4 with increasing DBP concentration can be attributed to the early loss of chlorophyll and partial uncoupling of the antenna system; when antenna size or effective absorption cross-section declines, basal fluorescence (Fo) decreases because less excitation reaches open reaction centers (RCs) [52,79,92]. By Day 8, however, Fo increased markedly at 8–10 mg/L, indicating a shift from antenna bleaching to genuine RC closure and damage involving the D1 protein and/or oxygen-evolving complex (OEC) [55,93]. The simultaneous rise in Fo and decline in Fm suggest that fewer RCs remained functional for charge separation and that the remainder were partially inactivated (Q_A_ reduced or donor-side limitation), consistent with progressive photoinhibition [51]. The steep fall in Fm and Fv/Fo at higher doses demonstrates impaired primary photochemistry and diminished efficiency of the water-splitting complex [94]. The strong, concentration-dependent rise in DIo/CSm and Fo/Fm reflects an increase in non-photochemical energy loss pathways. In early stress, this can be an adaptive photoprotective response (NPQ) that safely dissipates excess excitation as heat [95].

Leaf-level fluxes further confirmed these impairments. Reductions in TRo/CSm and ETo/CSm, together with increased DIo/CSm, indicate that photon trapping and electron transport per illuminated area declined while non-photochemical energy dissipation rose. These effects reflect chemical or oxidative modification of PSII core proteins (D1/D2), inhibition of plastoquinone reduction at the Q_B_ site, and decreased PQ-pool size or redox buffering [96,97]. The near-complete flattening of the J–I–P rise at high DBP supports the conclusion that electron flow beyond Q_A_ was severely restricted, creating a bottleneck and preventing normal charge turnover. Early increases in DIo/CSm likely represent regulated thermal dissipation (NPQ) [95], but sustained elevation of DIo accompanied by declining TRo/CSm and ETo/CSm by Day 8 denotes a transition from protective dissipation to irreversible photoinhibitory loss [98,99].

Because DBP is lipophilic, it readily partitions into thylakoid membranes, altering membrane fluidity, disturbing PSII–LHCII coupling, and promoting lipid peroxidation, as supported by elevated MDA levels. These perturbations weaken antenna–RC connectivity, hinder PQ diffusion, and compromise OEC integrity [100]. Oxidative modification of PSII proteins and inhibition of D1 turnover further lead to the accumulation of non-functional RCs [101,102]. The biophysical signatures—declines in ABS/CSm, TRo/CSm, and ETo/CSm with concurrent increases in DIo/CSm and Fo/Fm—align with biochemical evidence of pigment loss and lipid peroxidation [77,92]. Collectively, these processes curtail ATP and NADPH formation, reduce carbon fixation and biomass, and impair N-fixation capacity in *A. pinnata*, with direct repercussions for nutrient cycling and freshwater ecosystem stability.

Energy-flux analyses confirmed that DBP reduced both the density and functional capacity of active RCs. By Day 8, even 2 mg/L DBP lowered photochemical fluxes, while 6–10 mg/L caused sharp declines in RC/CSm, TRo/CSm, and ETo/CSm, coupled with pronounced increases in DIo/CSm. Loss of RC/CSm suggests inactivation or degradation of PSII units via D1 and OEC damage [55,103]. Suppression of TRo/CSm and ETo/CSm corresponds to reduced PQ-pool function and impaired Q_A_–Q_B_ turnover [75,97]. The dominance of DIo/CSm at Day 8 marks irreversible photoinhibition and the collapse of photoprotective capacity [51,104]. These findings parallel reports of phthalate-induced PSII impairment in aquatic macrophytes [105], indicating both structural loss and functional inefficiency that together depress primary productivity and nutrient recycling [106,107].

### 4.4. Quantum Yield Decline, Performance Indices, and Photochemical Inefficiency

Quantum-yield parameters provided further insight into energy partitioning. Under short-term exposure (Day 4), фPo and фEo remained relatively stable up to moderate concentrations, whereas фDo rose at 8–10 mg/L, reflecting early activation of thermal dissipation [71,75,108]. By Day 8, фPo and фEo declined sharply, and фDo increased, revealing progressive damage to PSII RCs, restricted electron flow through the PQ pool, and possible PSI acceptor limitation [104]. At this stage, energy dissipation reflects irreversible photoinhibition rather than regulated photoprotection [51,109]. Comparable declines in quantum yields with elevated dissipation have been documented in macrophytes such as *Lemna minor* and in algae, confirming similar redox disruption mechanisms despite organismal differences [77,109]. For *A. pinnata*, this shift from efficient photochemistry to sustained dissipation indicates a pronounced deficit in ATP/NADPH production, restricting photosynthetic carbon assimilation and nitrogen fixation and thereby weakening its role in water-quality regulation [110].

Performance indices (PIabs, PIcs) integrate light absorption, energy trapping, and electron transport [52,96]. DBP exposure caused progressive suppression of these indices and of derived ratios PHIo/(1–PHIo) and PSIo/(1–PSIo), particularly at higher concentrations and longer exposure [111,112]. A transient stimulation at 2–6 mg/L on Day 4 suggests a mild hormetic response [75,92,104], but by Day 8, PI values dropped sharply across all treatments, approaching zero at 8–10 mg/L. The collapse of PIabs indicates simultaneous impairment of antenna connectivity, RC density, and PQ-pool function [55,78,97,103]. Declines in PHIo/(1–PHIo) and PSIo/(1–PSIo) at 8–10 mg/L confirm that inhibition extended beyond PSII, affecting the entire photosynthetic electron-transport chain [51,104,112,113]. DBP’s membrane affinity and oxidative action elevate ROS levels and lipid peroxidation, causing irreversible PSII inactivation; by Day 8, repair mechanisms such as D1 turnover were overwhelmed [103,113]. Because PI values correlate strongly with plant vitality, their collapse forecasts major losses in carbon fixation and nitrogen assimilation, endangering *A. pinnata* ecological roles in biofertilization and nutrient regulation [76,104].

### 4.5. Multivariate PCA and Correlation Analysis as Diagnostic Indicators of DBP Stress

Multivariate analyses supported these interpretations. PCA distinctly separated high-dose (6–10 mg/L) treatments from controls, with Fo and фDo emerging as principal indicators of DBP stress, while controls clustered with PI, TRo/CSm, and ETo/CSm, signifying stable photochemistry. Greater separation on Day 8 emphasized the cumulative nature of toxicity [76,104]. Correlation analyses showed Fm, Fv/Fo, TRo/CSm, and ETo/CSm positively associated with PI, whereas Fo, DIo/CSm, and фDo were negatively correlated, reflecting a transition from efficient energy use to non-photochemical loss and confirming irreversible photoinhibition [104,109]. Together, these results demonstrate that DBP toxicity acts primarily by enhancing energy dissipation at the expense of photochemical conversion, validating chlorophyll-fluorescence parameters as sensitive biomarkers of phthalate stress in aquatic macrophytes, while acknowledging mechanistic parallels but physiological distinctions relative to algal systems.

**Figure 12:**
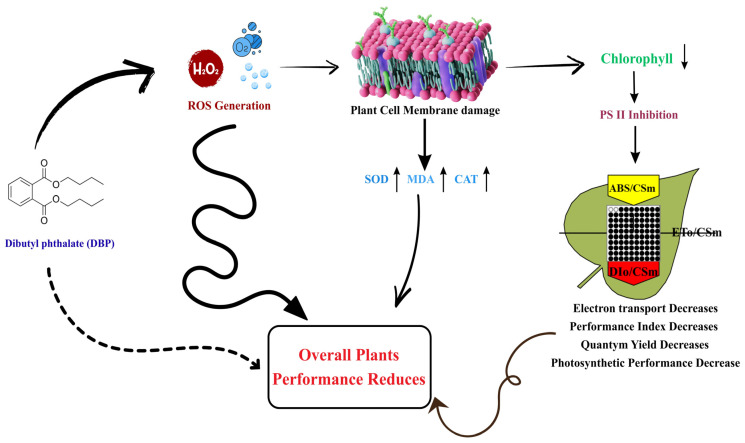
Proposed schematic representation of DBP-induced oxidative stress in *Azolla pinnata*, showing ROS generation, membrane damage, chlorophyll reduction, PSII inhibition, antioxidant enzyme changes, and decreased photosynthetic efficiency and plant performance.

The overall mechanism of DBP-induced oxidative stress, membrane lipid peroxidation, and subsequent inhibition of PSII activity in *A. pinnata* is summarized in Figure 12.

## 5. Conclusions

This study demonstrates that di-n-butyl phthalate (DBP) imposes severe, dose- and time-dependent toxicity on *A. pinnata* by disrupting both biochemical and biophysical processes central to photosynthesis. DBP exposure led to pigment degradation, enhanced lipid peroxidation, and an initial but unsustainable activation of antioxidant defenses, indicating progressive oxidative stress. Chlorophyll fluorescence analyses revealed that DBP impaired PSII functionality at multiple levels: elevating basal fluorescence, reducing maximal photochemical capacity, blocking electron transport, and ultimately collapsing performance indices. Energy flux mapping and quantum yield parameters confirmed a shift from efficient energy utilization toward excess energy dissipation, reflecting the transition from short-term photoprotection to long-term photoinhibition. Multivariate analyses further highlighted Fo and PHI(Do) as key markers of DBP-induced stress, while strong negative correlations between dissipation parameters and performance indices underscored the trade-off between energy loss and photochemical efficiency. Taken together, these findings provide mechanistic evidence that DBP compromises photosynthetic integrity and metabolic stability in *A. pinnata*, threatening its ecological role as a nitrogen-fixing and water-purifying species in freshwater systems. The results also reinforce the utility of chlorophyll fluorescence and antioxidant biomarkers as sensitive tools for assessing phthalate-induced stress in aquatic plants. Considering the widespread occurrence of phthalates in aquatic environments, the observed impairments highlight potential ecological risks and emphasize the need for stricter regulation and monitoring of these pollutants. Future studies should explore the recovery potential of *A. pinnata* under reduced contaminant load and evaluate community-level impacts to better predict the ecological consequences of chronic DBP contamination.

## Figures and Tables

**Table 1:** Summary of chlorophyll fluorescence parameters derived from OJIP transients, encompassing energy fluxes, quantum yields, and performance indices [47,48,53].

S.N.	Dynamics	Symbol	Formula	Definitions
**Fluorescence Parameters**				
1.	**Minimal fluorescence**	Fo	F_0_ ≅ F_50μs_	The minimum fluorescence signal was recorded at nearly 50 μs with the PEA fluorimeter, whereas the Handy-PEA instrument detected it earlier, at around 20 μs.
	**Maximal fluorescence**	Fm	Fm ≅ F_P_	The maximum fluorescence level associated with the P-step of the OJIP transient.
	**Variable Fluorescence**	Fv	Fm − Fo	The potential or peak variable fluorescence component that indicates the photochemical capacity of PSII.
**Density of Active PSII RCs per Cross-Section **				
2.	**Density of active PSII reaction centres per cross-section**	RC/CSm	$\frac{RC}{CS}={\varphi P}_{O}\cdot \left(\frac{V_{j}}{M_{O}}\right)\cdot (ABS/CS)$	Density of operational PSII centres relative to a unit of excited cross-section.
**Quantum Efficiency Parameters**				
3.	**Quantum yield for primary photochemistry**	PHI(Po)	$PHI(Po)=\frac{TR}{ABS}=\left[1-\left(\frac{F_{O}}{F_{M}}\right)\right]$	The highest quantum yield of primary photochemical reactions.
4.	**Quantum yield of electron transfer**	PHI(Eo)	$PHI(Eo)=\frac{ET}{ABS}=\left[1-\left(\frac{F_{O}}{F_{M}}\right)\right]\cdot \psi_{O}$	Quantum efficiency of electron transport beyond Q_A_^−^
5.	**Quantum yield of dissipation**	PHI(Do)	$\mathrm{PHI}(\mathrm{Do})=\mathrm{DI}/\mathrm{ABS}=F_{O}/F_{M}$	Quantum yield of excitation energy dissipated as heat and fluorescence
**Specific energy fluxes**				
6.	**Absorption per reaction centre**	ABS/CSm	$\frac{ABC}{RC}=M_{O}\cdot \left(\frac{1}{V_{j}}\right)\cdot \left(\frac{1}{{\varphi P}_{O}}\right)$	Effective absorption load borne by each active PSII reaction centre
7.	**Trapping per reaction centre**	TRo/RC	$\frac{TRo}{RC}=M_{O}\;\times \;\frac{1}{V_{j}}$	Flux of absorbed photons that are effectively trapped and drive primary charge separation at one RC
8.	**Electron transfer per reaction centre)**	ETo/RC	$\frac{ETo}{RC}=M_{O}\cdot \left[1/\frac{F_{2ms}-F_{O}}{F_{M}-F_{O}}\right]\cdot \Psi_{O}$	Electrons captured through photochemistry and transferred beyond Q_A_, expressed per active PSII reaction centre.
9.	**Dissipation per reaction centre**	DIo/RC	$\frac{DIo}{RC}=\left(\frac{ABS}{RC}\right)-[M_{O}\;\times \;\left(\frac{1}{V_{J}}\right)]$	Non-photochemical dissipation of energy (as heat or fluorescence) expressed per reaction centre.
**Phenomenological energy fluxes**				
10.	**Absorption per cross-section**	ABS/CSm	$\frac{ABS}{CSm}=Fluorescence\;intensity\;at\;50\;µs$	Photon absorption per unit illuminated cross-section, representing the total excitonic input to PSII antenna pigments.
11.	**Trapping per cross-section**	TRo/CSm	$\frac{TRo}{CSm}={\Phi P}_{O}\times \left(\frac{ABS}{CS}\right)$	Excitation energy successfully captured and used for primary photochemistry, expressed per unit cross-section.
12.	**Electron transfer per cross-section**	ETo/CSm	$\frac{ETo}{CSm}={\Phi P}_{O}\;\times \;\Psi_{O}\;\times \;\left(\frac{ABS}{CS}\right)$	Electron flux beyond Q_A_^−^ normalized to a cross-section, indicating PSII’s areal electron transport capacity.
13.	**Dissipation per cross-section**	DIo/CSm	$\frac{DIo}{CSm}=\left(\frac{ABS}{CS}\right)-[{\Phi P}_{O}\;\times \;\left(\frac{ABS}{CS}\right)]$	Proportion of absorbed energy dissipated as heat or fluorescence per cross-section, representing non-photochemical energy loss on an areal basis.
**De-excitation rate constants of PSII antenna**				
14.	**Non-photochemical de-excitation rate constant**	Kn	$K_{n}=K_{f}\;\times \;\left(\frac{ABS/CS}{F_{m}}\right)$	The rate constant for non-photochemical energy dissipation, with *Kf* representing the corresponding constant for photon re-emission as fluorescence.
15.	**photochemical de-excitation rate constant**	Kp	$K_{p}=K_{f}\;\times \;\left(\frac{ABS}{CS}\right)\;\times \;\left[\frac{1}{F_{o}}-\frac{1}{F_{M}}\right]$	Photochemical quenching (Kp) refers to the process by which absorbed light energy in chlorophyll is used for photochemistry in photosystem II, mainly driving electron transport. It reflects the efficiency of open PSII reaction centers in utilizing excitation energy for photosynthesis.
**De-excitation rate constants of PSII antenna**				
16.	**Performance index on an absorption basis**	PIabs	${PI}_{ABS}=\frac{1-(F_{O}/F_{M})}{M_{O}/V_{j}}\;\times \;\frac{F_{M}-F_{O}}{F_{O}}\;\times \;\frac{1-V_{j}}{V_{j}}$	An integrated parameter reflecting the potential efficiency of excitonic energy conversion into electron flow past Q_A_ into the intersystem electron transport pathway.
17.	**Performance index on cross section basis**	PIcsm	$\begin{array}{c} {PI}_{CS}=\frac{ABS}{CS}\;\times \;\frac{1-{(F}_{O}/F_{M})}{M_{O}/V_{j}}\;\times \;\frac{F_{M}-F_{O}}{F_{O}}\\ \;\times \;\frac{1-V_{j}}{V_{j}} \end{array}$	Performance index per cross-section, combining light absorption, excitation trapping, and electron transport efficiency.

## Data Availability

The original contributions presented in this study are included in the article. Further inquiries can be directed to the corresponding author.
